# Clinical features and progress in diagnosis and treatment of amyotrophic lateral sclerosis

**DOI:** 10.1080/07853890.2024.2399962

**Published:** 2024-12-03

**Authors:** Dongxiang Yuan, Shishi Jiang, Renshi Xu

**Affiliations:** Department of Neurology, Jiangxi Provincial People’s Hospital; The Clinical College of Nanchang Medical College; The First Affiliated Hospital of Nanchang Medical College; Xiangya Hospital of Center South University, Jiangxi Hospital; National Regional Center for Neurological Disease, Honggutan District, Nanchang, Jiangxi Province, China

**Keywords:** Amyotrophic lateral sclerosis, epidemiology, genetics, clinical features, diagnosis, treatment

## Abstract

Amyotrophic lateral sclerosis (ALS) is a fatal neurodegenerative disease of the central nervous system. Despite a large number of studies, the current prognosis of ALS is still not ideal. This article briefly describes the clinical features including epidemiology, genetic structure and clinical manifestations, as well as the progress of new diagnostic criteria and treatment of ALS. Meanwhile, we also discussed further both developments and improvements to enhance understanding and accelerating the introduction of the effective treatments of ALS.

## Introduction

**Figure 1.**
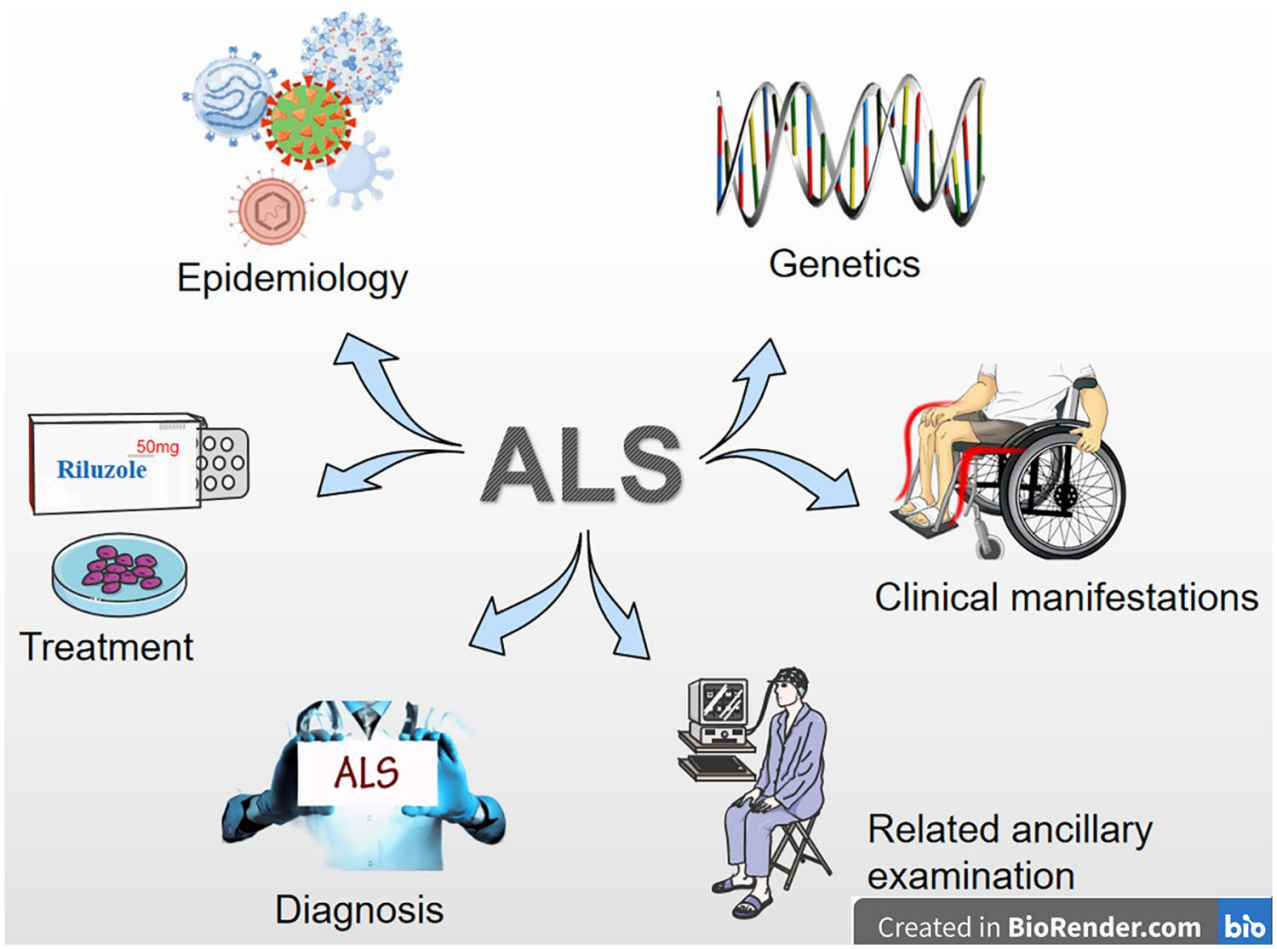
ALS: Epidemiology, genetic structure, clinical manifestations, diagnostic criteria and treatment.

Amyotrophic lateral sclerosis (ALS) is a fatal neurodegenerative disease of the central nervous system, it is a rare disease and is difficult to recognize, especially in its early stages [1]. Identifying the phenotypic and clinical heterogeneity and gaining a clearer understanding of the multisystemic damage nature of ALS, including cognitive dysfunction and behavioural changes, can help physicians better identify it earlier in the course of the disease. The development of new diagnostic methods, diagnostic criteria and different treatments as well as a clearer understanding of the course of ALS disease by physicians have a significant impact on the clinical diagnosis and treatment decisions and give a great hope for improving the prognosis of ALS patients. This review will provide an overview of these topics and current clinical practices in ALS patients (Figure 1).

## Epidemiology

ALS is a rare disease with age, gender, racial and geographic differences in incidence and prevalence. Some studies show that the incidence of ALS has remained stable over the past two to three decades, while others report a possible increase in incidence, which is expected to increase as the population ages [2]. Changes in the morbidity increase may result from improvements in diagnosis or changes in reporting standards, so the establishment of well-managed population registries is advocated. The most recent US-based ALS National Registry data show that the annual incidence of ALS ranges from 0.5/100,000 to 20.2/100,000 by age with a prevalence of 5.2/100,000, and that overall white people, men, non-Hispanics, people ≥60 years of age, and the people with a family history of ALS are more likely to develop ALS disease [3]. By the meta-analysis, the standardized global annual incidence of ALS was only 1.68/100,000, and the incidence also varied by gender with an overall standardized male-to-female ratio of 1.41 [4]. It also varied by region, in the populations of predominantly European origin, such as the Europe and the North America, the prevalence rates are slightly higher than the global average, ranging from 1.71/100,000 to 1.89/100,000, and possibly even higher in the population-based studies. The Asian populations have lower prevalence rates, ranging from 0.73/100,000 in the South Asia to 0.94/100,000 in the West Asia, while the highest prevalence rates are prevalent in the Oceania, 2.56/100,000 [4,5].

The incidence and prevalence of ALS in the different continents exist a larger variation. Among them, the average annual crude incidence is 1.01–1.22/100,000/year in Africa. In Asia, it ranges from 0.42/100,000/year in Iran to 2.20/100,000/year in Japan, the markedly higher incidence rate of 6.42/100,000/years was reported in Kii Peninsula and 23.46/100,000/year in Oshima Japan. The point prevalence of ALS was reported from 1.57/100,000 in Iran to 8.10/100,000 in Israel and was reported extreme values with the point estimates of 73/100,000 and 133/100,000 in the southern coastal regions of Papua Indonesia based on the relatively few studied populations. Similarly, it was reported a prevalence of 109.53/100,000 upper motor neurons (UMNs) disease and 1,010/100,000 lower motor neurons (LMNs) disease in Gujarat India. The period prevalence estimates over various time frames ranged from an average annual prevalence of 1.97/100,000/over 3 years in Taiwan to one year period prevalence of 9.90/100,000 in Japan. The prevalence in South Korea over one-year period ranged from 3.43/100,000 to 6.49/100,000.

In Europe, overall, the average annual crude incidence ranges from 1.11/100,000/year in Serbia to 5.55/100,000/year in Denmark and from 1.33/100,000/years in Sardinia to 3.22/100,000/years in Liguria in Italy. Most studies report that the incidence rate is higher men than women, a peak incidence is at 70–79 years of age and declines thereafter. The lowest incidence rate in all European studies was reported in Serbia, Russia and Cyprus. The highest incidence rate was reported in Scotland, Austria and Denmark. Overall, the prevalence studies in Europe were reported that the point prevalence ranged from 3.44/100,000 in Malta to 10.80/100,000 in Italy. In North America, the average annual crude incidence ranged from 0.5 to 3.29/100,000/year in Canada, from 1.08/100,000/years to 2.20/100,000/year in United States. The point prevalence of ALS in North America ranged from 2.00/100,000 to 11.80/100,000 in United States.

In the South America, the average annual crude incidences were reported were 0.26/100,000/year in Ecuador, to 1.40/100,000/year in Colombia and 3.17/100,000/year in Argentina. Point prevalence was 4.90/100,000 in Colombia, 5.00/100,000 in Brazil, to 8.86/100,000 in Argentina. In Oceania, incidence was estimated at 2.9/100,000/year in New Zealand.

ALS is a rare neurodegenerative disorder mainly affected UMNs and LMNs. The epidemiological investigation of ALS is an inherent challenge because of the rarity and the rapid progression nature of ALS, which is difficult to overcome without the adequate clinical and research resources. Some resources are not readily available everywhere in worldwide, which results in difference in the information concerning the epidemiology of ALS in some regions. Most epidemiology investigations of ALS were conducted in Europe and North America. Although the epidemiology investigations were fewer, the difference was also reported in some remained regions such as Africa, Oceania and South America. Therefore, there was variability in the estimated incidence and prevalence reported both within and between countries and continents based on the available data. The reasons about these differences have not been fully explored now. Firstly, researcher is required to directly contact to the epidemiological investigators to exchange the details of epidemiological investigation methodology in many cases in order to analyse the epidemiological investigation data, which is very difficult in the worldwide. Secondly, the possible reasons are differences in the certainment, coverage and representativeness of targeted populations, and genetic and/or environmental factors both within and across the geographical regions. Besides, it is unlikely that major differences are the usage of different diagnostic criteria because most studies used the Original or Revised El Escorial criteria [6].

## Genetics

ALS is currently classified as the familial or the sporadic ALS. The majority of ALS patients have no known family history of ALS, around 10% of ALS patients with the familial characteristics are the familial ALS (fALS), fALS has a history of ALS in family members with the autosomal dominant, the autosomal recessive, the X-chromosomal inheritance and the high ectopic rates [7,8]. Approximately 70% of fALS and almost 15% of sporadic ALS (sALS) onset can be explained by mutations in the known pathogenic genes [9]. In additional, the sporadic cases with low penetrance mutations, and the fALS belonging to small families or with the incomplete or insufficient information of family history may actually be fALS, the under-reported fALS may account for approximately 20% of fALS cases [10].

**Figure 2.**
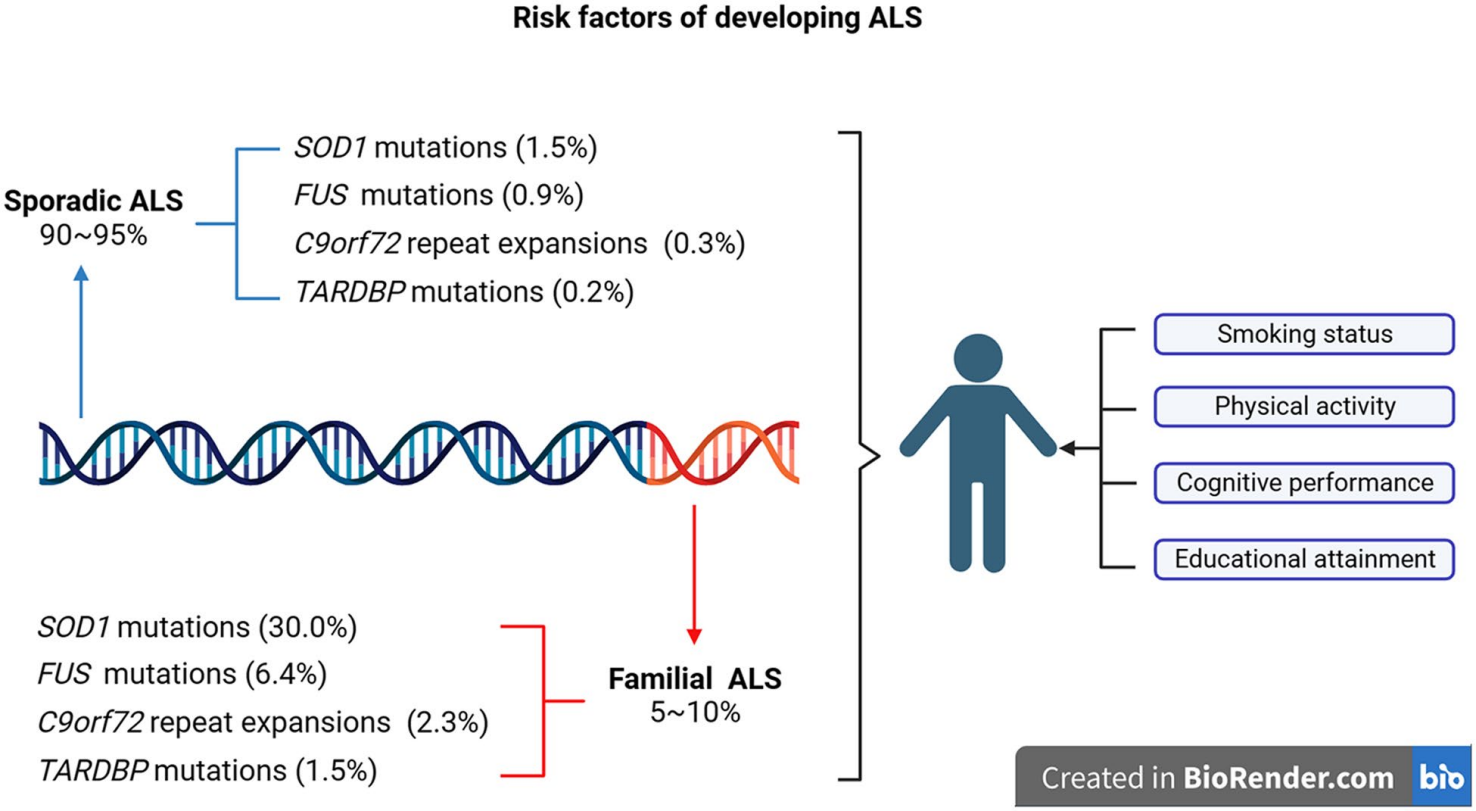
Risk factors of developing ALS: in the asian populations, four main ALS related mutation genes were *SOD1* (fALS 30.0%, sALS 1.5%), *FUS* (fALS 6.4%, sALS 0.9%), *C9orf72* (fALS 2.3%, sALS 0.3%) and *TARDBP* (fALS 1.5%, sALS 0.2%). polygenic risks for ALS with traits and single nucleotide polymorphisms seem to be associated with the smoking status, the physical activity, the cognitive performance and the educational attainment.

Although the pathophysiological mechanisms of ALS are not fully understood, the influence of genetic factors regarding ALS has been widely recognized in recent years with the extensive use of genome-wide association studies and sequencing technologies and the rapid development of molecular biology. The genetic architecture of ALS is highly complex and bases mainly on the single-gene inheritance of rare variants. At present, more than 40 ALS-related causative genes have been identified [11,12]. The most common pathogenic genes among them are *C9orf72*, *TARDBP (TDP-43)*, *SOD1* and *FUS* although the frequency of genetic isoforms varies according to the population ancestry. According to Zou’s study, in the Asian fALS population, about 30% of cases are *SOD1* mutations, 6.4% of cases are *FUS* mutations, 2.3% of cases are *C9orf2* mutations, and *TARDBP* mutations account for 1.5% of cases (Figure 2) [13]. Compared to fALS patients, the proportion of these ALS-related genes involved in pathogenesis among sALS patients is very lower. In addition to the primary monogenic inheritance of ALS, the impact of oligogenic and polygenic inheritance on the ALS disease risk also has received attention. Several studies have highlighted that the genetic screening has identified a subset of ALS patients that contain two or more variant ALS genes, which may play a role in the ALS risk and progression [14,15]. The genetic profiling identifies a common polygenic risk for ALS with traits and single nucleotide polymorphisms associated with the smoking status, the physical activity, the cognitive performance and the educational attainment (Figure 2) [16,17].

Various genetic studies report that *C9orf72*, *SOD1*, *TARDBP* and *FUS* are the currently found commonest mutative genes in ALS. A meta-analysis reported that the overall pooled mutation frequency of these commonest ALS pathogenic genes was 47.7% in fALS. It was found that there was a significant difference in the mutation frequency of these major ALS-related genes between the European and Asian populations. In the European ALS patients, the commonest mutations were the *C9orf72* GGGGCC repeat expansions in fALS, among them, 33.7% of cases *C9orf72*, 14.8% of cases *SOD1*, 4.2% of cases *TARDBP* and 2.8% of cases *FUS* respectively. While in the Asian ALS patients, the commonest mutations were *SOD1* mutations in fALS, among them, 30.0% of cases *SOD1*, 6.4% of cases *FUS*, 2.3% of cases *C9orf72* and 1.5% of cases *TARDBP* mutations respectively. These findings demonstrate that the related genetic mutations of ALS in the Asian ALS patients is distinct from that in the European patients, which need 06appropriately consider this di2fference when ALS patients are performed the genetic test [13].

The *SOD1* mutations cause approximate 12–25% of fALS cases, and the intra-familial clinical phenotypes caused by the *SOD1* mutations are usually heterogeneous and variable. The L144S *SOD1* mutation was identified in Brazil, Iran and United States, and also is the second commonest *SOD1* mutation in the Polish ALS patients. The *SOD1* L144S mutation displays the geographically different clinical phenotypes in the distinct populations from the different regions of worldwide. The clinical presentation of the Polish ALS patients of *SOD1* L144S mutation exhibits a relatively slow uniform course, a prevalent onset in the lower limbs, either classic or progressive muscular atrophy presentation, a long survival time, an intra-familial lower heterogeneity and penetrance. 18.3% of *C9orf72*, 12.2% of *SOD1*, 5% of *FUS*, 3.7% of *TARDBP*, and 2.4% of *UBQLN2* mutation frequencies were reported in the Turkish fALS patients respectively [18]. In Southern Africans, five different N87S, G94D, I114T, L145S, and L145F SOD1 variants in five individuals of one familial case were detected, and the pathogenic C9orf72 GGGGCC repeat expansions were detected in seven individuals of another familial case [19].

The *SOD1* mutations produce the insoluble *SOD1* ubiquitin-positive inclusion body in the motor neurons (MNs) of ALS patients, which interferes the folding and synthesis and removal of proteins as well as proteasome degradation, leading the degradation dysfunction of abnormal and toxic proteins as well as the misfolding and aggregation of proteins such as mutative SOD1 in ALS. The misfolding and aggregating proteins result in the endoplasmic reticulum stress and the ubiquitin-proteasome system (UPS) activation to generate a vicious circle of abnormal toxic proteins production. Besides, the toxicity of mutant *SOD1* also damages the removal of abnormal aggregates by autophagy, which further produces the excessive aggregation and deposition of pathological proteins including the mutated SOD1 to generate the insoluble *SOD1* ubiquitin-positive inclusion body, creating a vicious cycle that leads to cell death in the pathogenesis of ALS.

The hundreds or thousands of GGGGCC nucleotide repeat expansion in the intron of *C9orf72* gene drives the pathogenesis of ALS through the loss of the normal function and the toxic effects acquisition of *C9orf72*, the RNA amplification, the dipeptide repeat proteins aggregation and the *C9orf72* haploid dysfunction, which produces the cytotoxic to interfere and affect the protein degradation *via* impairing UPS, producing the ubiquitin-positive characteristic pathological neuronal inclusions. Moreover, the *C9orf7*2 mutant selectively inhibits the proteasome subunit to induce the death of MNs, and the abnormal proteasome is presented in the inclusion body, which further supports that the *C9orf72* mutation induces the occurrence and development of ALS by interfering with UPS. In addition, autophagy also might lead to the imbalance of protein homeostasis in neurons by the degradation decrease of abnormal toxic proteins due to the dysfunction of autophagy.

The neurocytotoxicity induced by the *TDP-43* gene mutation has been widely recognized as one major pathological factor in the pathogenesis of ALS. *TDP-43* involves in lots of cellular functions, such as gene transcription and RNA processing. The cytoplasmic TDP-43 produced by *TDP-43* mutation involves in the accumulation and deposition of misfolded proteins in brain through inhibiting and/or impairing the UPS, proteasome and autophagy activity in the *TDP-43* mutation ALS patients, which results in the degradation decrease of pathogenic proteins including FUS protein. In addition, The *FUS* mutations mainly lead to the FUS mislocalization in cytoplasm, which leads to the production of FUS immunoreactive inclusion bodies leading to the neurons degeneration in the pathogenesis of ALS [20].

## Clinical manifestations

**Figure 3.**
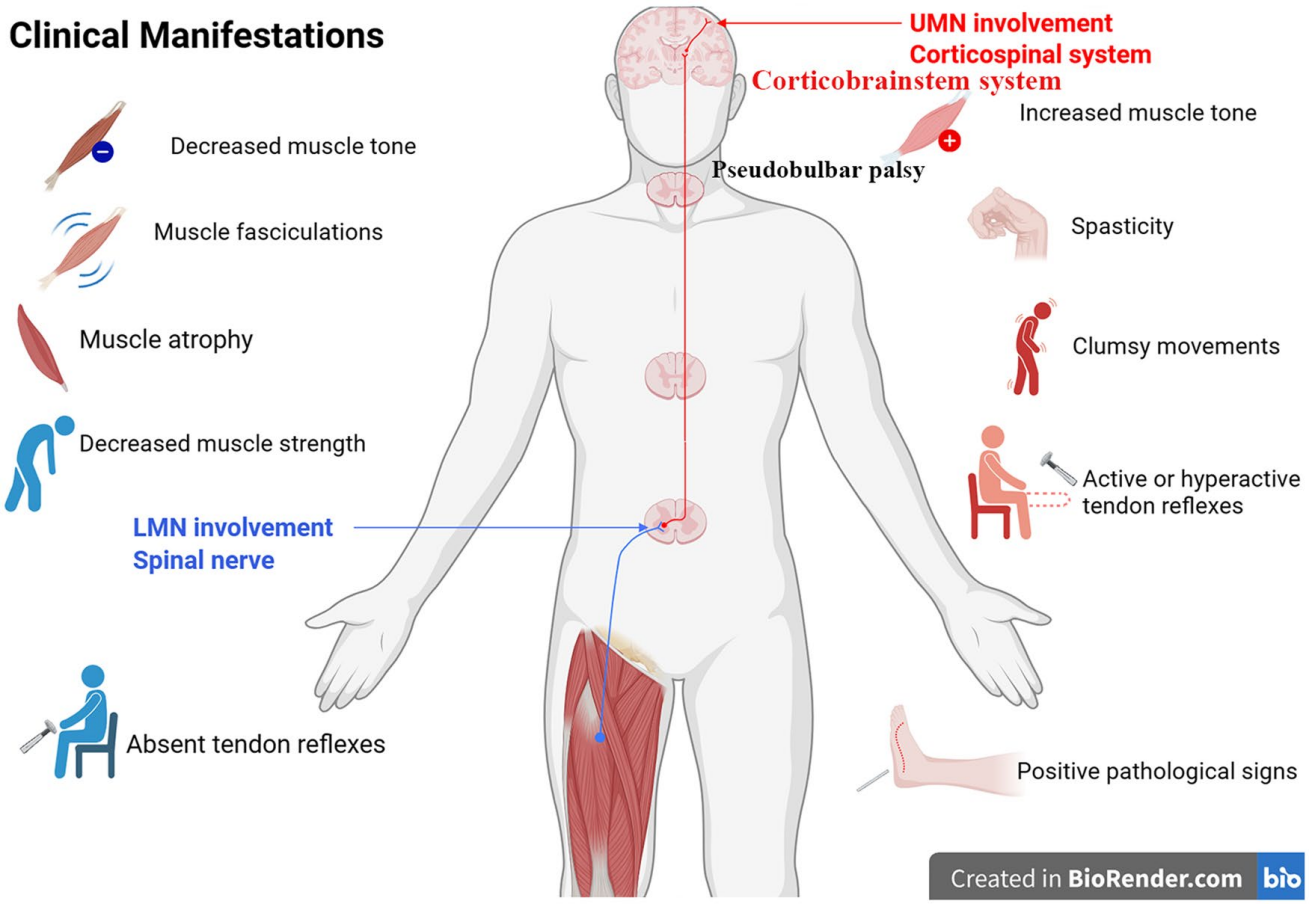
Clinical manifestations: the clinical manifestations of UMNs and LMNs damage, ALS patients progressively exhibit the appropriate clinical symptoms. The clinical manifestations of UMNs dysfunction contain the increased muscle tone (spasticity), the active or hyperactive tendon reflexes, the positive pathological signs, the pseudobulbar palsy and the clumsy movements. The clinical manifestations of LMNs dysfunction contain the decreased muscle tone, the muscle atrophy, the decreased muscle strength, the muscle fasciculations and the diminished or absent tendon reflexes.

ALS is mainly characterized by the motor symptoms and signs of UMNs and LMNs damage involving the bulbar, the spinal cervical, thoracic or lumbosacral segments. The UMNs damaged clinical manifestations mainly exhibit the increased muscle tone (spasticity), the active or hyperactive tendon reflexes, the positive pathological signs, the pseudobulbar palsy and the clumsy movements. The LMNs damaged clinical manifestations mainly display the decreased muscle tone, the muscle atrophy, the decreased muscle strength, the muscle fasciculation and the diminished or absent tendon reflexes (Figure 3) [21].

An investigation of 470 patients with ALS found that almost 85% of ALS patients developed from one side of spinal cord segment to the opposite side, then to the adjacent anatomical segments and/or the non-adjacent segments [22]. ALS manifests in a variety of phenotypes, and there is no unified typing standard for ALS due to lacking biomarkers for the effective classification. The most common clinical typing method for ALS is based on the involvement degree of UMNs and LMNs. They are classified as the classic ALS limb onset, the classical ALS bulbar onset, the flail arm syndrome, the flail leg syndrome, the primary lateral sclerosis, the progressive muscular atrophy, the ALS combined with frontotemporal dementia (FTD, cognition onset ALS), the isolated bulbar palsy [23]. ALS presents as multiple phenotypes, among them, the bulbar onset and spinal cord onset (cervical and lumbar segments) ALS are the most common presentations, each constitutes about a quarter of ALS cases. The respiratory failure onset, or the hemilateral flail arm and leg, the hemilateral primary lateral sclerosis and progressive muscular atrophy is less frequent [24,25]. ALS phenotypes are influenced by age, sex and genetics, the women of 60 years or older are more likely to have a classical ALS bulbar onset phenotype, whereas the men under 60 years are more likely to exhibit a classic ALS limb onset phenotype, the pure UMNs onset phenotypes are more common in men and women under 60 years, and the onset of flail arm, flail leg and respiratory failure occurs predominantly in men independent of age. The *C9orf72* GGGGCC amplification is associated with the bulbar onset and ALS-FTD phenotypes, the *SOD1* mutation is associated with the limb onset (spinal cord onset) phenotype [26].

In addition to the typical motor symptoms, ALS also has some non-motor symptoms such as anxiety, depression, fatigue, pain, sleep disturbance and cognitive impairment [27]. The typical ALS is primarily considered as a disease of motor dysfunction such as dysarthria, dysphagia, and limb muscle weakness and atrophy. However, the cognitive and behavioural changes may occur early in the course of the ALS disease such as the decrease of the social cognitive abilities and the executive and verbal memory functions [28]. The cognitive dysfunction is a common feature of ALS and is now thought to occur in 35-50% of patients with ALS, which cannot be attributed by the physical, respiratory or emotional deterioration, because the physical, respiratory or emotional deterioration are similar between the cognitively stable patients and those with the progressive cognitive damaged patients [29,30]. In a follow-up of changes in the cognitive and behavioural deficits from diagnosis to 6 months in 146 patients with ALS, the cognitive deterioration was found in approximately 30% of ALS patients, even in those who initially presented with the normal cognitive abilities. The ALS patients with the cognitive decline more rapidly progressed clinically and had a shorter survival than the ALS patients with the normal cognition [30]. Approximately 15% of patients with ALS meet the diagnostic criteria for frontotemporal dementia among all ALS patients [31]. *TARDBP* is present in approximately 97% of patients with ALS and 50% of patients with frontotemporal dementia, which together with these cognitive and behavioural changes support the idea that ALS is on the same continuum as frontotemporal dementia [23]. The Edinburgh Cognitive and Behavioural ALS Screen (ECAS) is a validated, multidomain, assessment tool developed for patients with ALS, which can be administered by neuropsychological and non-neuropsychological professionals [32]. ECAS is a clinically extensively used screening tool composed of series of short cognitive test items and is determined to be sensitive to test the cognitive impairment of ALS patient. ECAS can estimate the executive functions, memory, language, the visuospatial skills and the social cognition, especially in the brief assessment of behaviour and psychosis can interview with the patients carers or relatives. ECAS can answer *via* verbal or combining writing or point, is suitable for patients who hand motor functions are completely destroyed. Total score of ECAS is 136 points and need no longer than 15 min to finish. Two alternate versions of ECAS B and C are developed for situations where serial testing is required. Specific guidance on the administration of ECAS B and C can be obtained at http://ecas.psy.ed.ac.uk/or contacting s.abrahams@ed.ac.uk.

Recently, an increasing number of studies have indicated that ALS is a multisystemic disorder with the widespread brain involvement. Thus, ALS patients may have multiple non-motor symptoms. In addition to cognitive deficits, other non-motor symptoms, such as anxiety, depression, fatigue, pain, sleep disturbance, are also common in ALS patients. A systematic review reported that ALS patients might exhibit the depression and anxiety symptoms at different levels. However, the studies assessed the depression and anxiety symptoms in ALS patients reported contradictory results. Therefore, it needs further study in larger ALS samples using the specific instruments measuring depression and anxiety symptoms in order to ascertain the depression and anxiety symptoms whether or not leading by ALS and their prevalence [33]. A systematic review and meta-analysis about the frequency and correlation of fatigue in ALS patients found that fatigue represents in approximately 50% of ALS patients and is related to the lower function status and the poorer quality of life, which implies that the assessment and management of fatigue in ALS patients are very important for improving function status and the quality of life for ALS patients [34]. Pain symptom in ALS patients is at large scale neglected, in fact, majority of ALS patients occur pain at whole disease stages, even may occur before the onset of motor symptom. Pain deteriorates the quality of life and increases the anxiety and depression prevalence of ALS. Moreover, pain may predict the clinical deterioration and death. The pain type often includes cramp pain, nociceptive pain or neuropathic pain [35]. The sleep disturbances in ALS patients may be related to the reduce mobility, muscle cramps, swallowing problems, anxiety, depression, restless legs and increased myoclonic activity. Moreover, the hypoventilation leading by sleep-disorder breath in ALS patients interferes the sleep patterns, which may result in daytime sleep symptoms (excessive daytime sleepiness) and affects the daily activities and further increases the incidence of anxiety and depression, producing the vicious circle of sleep disturbances [36].

## Electromyography

Electromyography has a higher diagnostic value and is an effective test for identifying clinical and subclinical LMNs damage. In the absence of valid biological diagnostic markers for ALS, electrophysiological examination, as an extension of clinical examination, plays an irreplaceable role in the diagnosis of ALS and provides objective evidence of LMNs damage in ALS [37]. Normally, the latency of motor nerve conduction terminals in ALS patients is normal or mildly prolonged, and conduction velocities are mostly normal or mildly slowed, but not below 70% of the normal baseline, while when the muscle atrophy is evident, there can be a reduction or disappearance of compound muscle action potential (CMAP) wave amplitude, especially in the weakness muscles. A mild slowing of motor nerve conduction velocity may occur when the CMAP amplitude is significantly reduced. The ALS motor nerve conduction tests may show a ‘split hand’ phenomenon because the thumb and the first interosseous muscles occur earlier atrophy and weakness and are more severely damage on the course of ALS, while the little finger adductor muscle is relatively preserved. Meanwhile, when the CMAP wave amplitude ratio of thumb/thumb adductor muscle is < 0.6 or the CMAP wave amplitude ratio of first interosseous muscle/thumb adductor muscle is < 0.9 is of the great value for the early diagnosis and differential diagnosis of ALS [38].

A study on the CMAP amplitude of motor nerve conduction in the lower extremities of ALS patients revealed that the so-called ‘split leg’ phenomenon also existed in the lower extremities, that is, the gastrocnemius muscle was more severely damaged than the anterior tibial muscle in ALS patients [39]. These signs were rare in the neurological and non-neurological disorders other than ALS. Sensory nerve conduction was usually normal in ALS, but some studies had suggested that mild sensory conduction abnormalities were mainly in the form of decreased sensory conduction wave amplitude, F waves were usually normal, but when there was a significant muscle atrophy, the corresponding nerve was seen to have a decreased F wave rate, while conduction velocity was relatively normal [40].

A retrospective analysis of clinical symptoms and electromyography diagnostic examinations in 150 patients with confirmed or suspected ALS revealed that the positive diagnostic rate of electromyography varied depending on the site of onset. The patients with the electromyography abnormalities seen in approximately 40% of asymptomatic limb muscles, with a higher sensitivity in distal limb muscles (78.4%) than in proximal limb muscles, and the highest sensitivity in cervical muscles (86.3%) [41].

Among the electrophysiological examinations, needle electrode electromyography is also very important in determining LMNs damage in ALS, mainly looking for the electrophysiological evidence of neurogenic damage in the active phase (positive sharp waves, fibrillation potentials and fascicular fibrillation potentials) and in the chronic phase (widening of motor unit potential time frame, increased wave amplitude and polyphasic waves). In the electromyography diagnosis, the muscles innervated by the four body regions of medulla oblongata, the cervical, thoracic and lumbosacral spinal cord are usually examined. In the cervical and lumbar spinal regions, at least 2 or more muscles innervated by the different nerve roots and the different peripheral nerves should be examined [42]. It is found that needle electrode electromyography abnormalities were highest in the distal limb muscles, including the first interosseous muscle, the short thumb, the anterior tibialis and the gastrocnemius in 354 patients with ALS, and were not related to the site of disease origin [43]. In the electromyography clinical practice, there are still difficulties in identifying the normal fasciculation potentials and the pathological fasciculation potentials, and the characteristics of the emergence of fasciculation potential waveforms, the frequency of delivery and interval time also need to be studied in depth. In general, we need to emphasize that ALS diagnosis is indeed a clinical process, with electromyography/nerve conduction examination serving as the crucial extensions of the physical examination. These tests are instrumental in confirming the presence of ALS by demonstrating the impairment of MNs. Other diagnostic tests, such as imaging, are primarily utilized to rule out conditions that may mimic ALS symptoms, ensuring an accurate diagnosis.

## Transcranial magnetic stimulation

As a non-invasive technique, the transcranial magnetic stimulation enhances our understanding of the pathophysiology of ALS through the motor threshold, the motor evoked potential (MEP) amplitude, the central motor conduction time, the cortical silent period, the intracortical inhibition and the facilitated assessment of motor cortex and cortical function. MEP after the transcranial magnetic stimulation can confirm the damage of UMNs or corticospinal pathways. ALS patients exhibit the cortical hyperexcitability, the increased motor thresholds and the impaired intracortical inhibition (especially short interval intracortical inhibition) in the examination of transcranial magnetic stimulation [44]. To date, the MEP of transcranial magnetic stimulation remains the test of choice for identifying the UMNs damage. A study in 176 ALS patients diagnosed between 2011 and 2014, reported that the MEP abnormalities were found in 80% of patients with the clinical evidence of UMNs damage and in 72% of patients without the clinical involvement of UMNs, in the latter, 61% of patients without the clinical involvement of UMNs showed the clinical signs of UMNs damage at 1 year, and approximately 70% of patients with the clinical LMNs damaged phenotype showed the MEP abnormalities [45]. The transcranial magnetic stimulation technique can detect the UMNs damage in ALS months in advance, and can partially identify ALS and the diseases involving UMNs [46]. The use of threshold tracking transcranial magnetic stimulation has shown utility in monitoring the disease progression of ALS, the homologous circuit dysfunction is associated with greater disability and a faster rate of disease progression [47].

## Neuroimaging

The magnetic resonance imaging (MRI) of cranial and spinal cord is an indispensable test in the diagnosis of ALS. While it does not provide a definitive diagnosis of ALS, it provides an important differential diagnosis of diseases similar to ALS. For example, the herniated disc, the tumor or malformation compressing both cervical nerve roots and spinal cord may lead to the LMNs symptoms in arms and the UMNs symptoms in legs and be misdiagnosed as the classical ALS [48].

A cranial magnetic resonance water suppression imaging study of 82 patients with clinically confirmed sALS was found that the high signal in the pyramidal tract projection area, especially in the precentral gyrus, is important to support the determination of UMNs damage. The magnetic resonance diffusion tensor imaging (DTI) can reflect the pathological changes of the UMNs degeneration or absence in the brain of ALS patients by detecting changes in the fractional anisotropy values and the mean diffusion coefficient values, and determine the neuronal involvement, especially when the clinical manifestations of UMNs damage are not obvious, but it is currently used only for a scientific research, the confident evidences need further and deeply investigate extensively in the multiple centres and large samples [49].

MRI is an important neuroimaging diagnostic method, widely uses in the differentiated diagnosis of ALS. MRI has lots of different imaging, such as T1- and T2-weighted imaging (TWI1 and TWI2), DTI, diffusion-weighted imaging (DWI), susceptibility-weighted imaging (SWI), resting-state functional MRI (rs-fMRI), magnetic resonance spectroscopy (MRS). These imaging play an important role in the diagnosis of ALS. Conventional MRI can detect brain and spinal cord construe alterations of ALS patients using TWI1 and TWI2, especially the gray matter thickness. DTI can identify white matter fibre damage of ALS patients *via* measuring the diffusion distance of water molecules in each gradient direction. MRS can observe metabolic changes in the brain of ALS patients through detecting the concentration of different chemicals. rs-fMRI can find the brain activity and functional connections between the different brain of ALS patients by measuring the blood-oxygen level-dependent signals. The MRI SWI is sensitive to the presence of paramagnetic material such as calcium and iron. Moreover, SWI is more sensitive to the recognize the motor band signs in ALS patients than TWI2 [50]. The ultra-high-resolution MRI can obtain more accurate and high contrast images, currently used mainly for a scientific research.

The studies of TWI1 or TWI2 found that regional cortical thinning on TWI1 was the definite feature of ALS, the reduction of cortical thickness also was observed in the motor (precentral gyrus) and extra-motor (frontotemporal) regions of cerebrum [51]. DWI and DTI revealed the extensive regional changes in brain dispersion indicators in ALS patients. MRS detected that the concentration of some chemicals such as N-acetylaspartate neurotransmitter significantly change in the motor cortex, the corticospinal tract and hippocampus [52]. The usage of MRI has largely facilitated the development of ALS diagnosis, especially in combining to use multiple MRI imaging. The network analysis of brain MRI showed that ALS patients exhibited the altered structural overall network properties in sensorimotor, basal ganglia as well as the frontal and parietal regions, specifically, the abnormal structural connections were associated with motor deficits, while the disrupted functional connections were consistent with changes in cognition and behaviour [53].

The glymphatic system recently is found to be an important clearance pathway in brain, playing a key role in regulating the brain metabolites such as glucose and lipids. It is noted that the glymphatic system involves in maintaining the protein homoeostasis possibly through multiple pathways such as the alteration of arterial pulsation, respiration, sleep and ageing. Therefore, it receives the researchers considerable attention in researching the neurodegenerative diseases, particularly Alzheimer’s disease. However, although ALS is also belong to proteinopathy, the researches about the relationship between glymphatic function and ALS are relatively insufficient yet at present [54]. The glymphatic system transports solutes and scavenges the toxic material such as the ALS pathogenic proteins TDP-43 and the excitatory neurotransmitter glutamate in the pathogenesis of ALS from brain *via* the meningeal lymphatic vessel. The glymphatic system is regulated by arterial pulsation, respiration, posture, sleep, as well as the position and proportion of aquaporin-4 channels. Non-rapid-eye-movement slow wave sleep is important to the glymphatic drainage discontinued during wakefulness. In Parkinson’s disease and Alzheimer’s disease, the sleep impairment is found early before several years of the onset of characteristic clinical features and is related to the progressive accumulation of toxic pathogenic proteins. Although the sleep issue in ALS patients has been confirmed by more and more investigation evidences, the pre-clinical sleep impairment or the possibility of glymphatic system impairment in ALS has fewer been investigated [55].

## Biofluid biomarkers

There are no valid biological markers to confirm the diagnosis of ALS, and the relevant laboratory tests are mainly used to exclude the ALS-like diseases. For the patients with the clinically suspected ALS, the necessary related auxiliary examinations should be performed such as blood sedimentation, creatine kinase, creatinine, uric acid, connective tissue, thyroid function, protein, tumor markers in blood and the cerebrospinal fluid (CSF) routine. The CSF examination is indispensable at the early stages of ALS to rule out other neurological disorders similar to ALS. The neurofilament light chain (NFL), TDP-43 and tau protein in blood and/or CSF may hold promise as the diagnostic biomarkers and predict the disease progression [56]. The elevated NFL concentrations in CSF and plasma have been reported to be associated with a shorter survival, a more aggressive disease phenotype, and the *C9orf72* amplification [57,58]. However, from the current data, NFL and the phosphorylated neurofilament heavy chain (pNFH) levels in the CSF and serum of patients with ALS significantly increased only at the onset of symptoms or during symptomatic episodes [59].

The large researches have showed that the neurofilaments levels in both CSF and blood significantly increase in ALS patients compared with that of healthy populations. Moreover, more and more evidences support neurofilaments as a potential diagnostic biomarker, and the clinically valuable biomarkers monitoring the disease progression and survival of ALS [60]. Neurofilaments are usually divided into NFH, the medium (NFM) and NFL based on the big and small of their molecular mass, they are one of the fundamental scaffolding protein of axons. Both NFM and NFH are necessary for the post-translational modifications such as O-glycosylation or phosphorylation. For the diagnosis of ALS, the NFL and the phosphorylated-NFH subunits are considered for the potential biomarkers [61].

The neurofilaments are one of the commonest gene mutative translated proteins in ALS [62]. The NFL in CSF or serum is helpful to the diagnosis and prognosis judgment of ALS patients, and the NFL contents in CSF or serum gradually increase with the increase of age [63] and the NFL levels in both CSF and serum significantly elevate in ALS patients. The NFL levels in serum were reported to be a good judged biomarker on the progression of ALS disease [64], and the NFH in both CSF and/or serum also play a similar role with NFL [65], the NFH levels in both CSF and/or serum are significantly correlated with the survival of ALS patients [62].

The contents of neurofilaments proteins did not change significantly at the different stages of ALS disease progression, which is suggested that the measurement of neurofilaments contents at the initial stage can provide the information about the speed of disease progression. The current studies showed that there was a significant negative correlation between the neurofilaments protein levels primarily in both CSF and serum and the survival prognoses of ALS [58,66–68].

At present, the studies in the fALS patients with the pathogenic variants of several ALS related genes found the difference of neurofilaments levels at the disease early stage compared with the familial members. Meanwhile, it is found that the elevated serum NFL levels in the fALS patients of the pathogenic *SOD1* A4V2 variant could be up to 12 months before the noticeable symptoms are appeared. The fALS patients of *C9orf72* variant could be early up to 2 years and fALS patients of the *FUS* variant could be up to 3.5 years [69].

The elevated NFL levels in the CSF of C9ORF72 carriers is closely linked to the GM volume reduction in the ventral and dorsal prefrontal cortices, the ventral and dorsal lobes, the anterior cingulate gyrus, caudate, the medial thalamus and the other frontotemporal parietal regions [70]. At present, the reliability of neurofilaments as a diagnostic biomarker in the judgement of onset location is very limited.

The neurofilaments are combined examination with other biomarkers such as pNFH, TDP-43, tau protein [71], interferonγ [64], Titin protein [72], microRNA, neuroinflammatory-related proteins, p75(NTR) extracellular domain, p-Tau/t-Tau, ubiquitin C-terminal hydrolase L1, extracellular vesicles [73] and muscle proteins [74]. The examination of neurofilaments in CSF or serum for the diagnostic sensitivity and specificity of ALS might improve compared with the sole test of neurofilaments.

Neuron axon damage is one of various neurodegenerative diseases features including ALS. pNFH is the cytoskeletal structural protein released into CSF because of axon damage, subsequently into blood. Because of the high specificity for neuron damage, pNFH is more advantage than other biomarkers for ALS disease diagnosis, is considered as a valuable and significant diagnostic and prognostic biomarker in ALS patients. High pNFH levels were detected in both fALS and sALS patients and were linked to the faster disease progression [75,76]. Both NFL and pNFH were associated with both UMN and LMN damage. However, NFL was better correlated with the UMN damage, whereas pNFH was better correlated with the LMN damage. Although the diagnostic and prognostic roles of NFL and pNFH in serum and/or CSF for ALS have a better promising, the elevated levels of NFL and pNFH in serum and/or CSF also are found in lots of other neurological disorders, such as multiple sclerosis, dementia, traumatic brain injury, stroke, atypical Parkinson’s disease, Huntington’s disease and bipolar disorder [60,77].

ALS currently exists the substantial heterogeneity of clinical phenotype, and the potential complex pathogenesis have not been completely clarified. Besides the genetic factor, the onset site and the onset age, the neuroinflammation roles also obtained more and more evidence for the ALS exacerbator and driver, among them, chitinases is particularly intriguing in the pathogenesis of ALS, the related studies reported that the chitotriosidase (CHIT1), chitinase-3-like-1 (CHI3L1) and chitinase-3-like-2 (CHI3L2) levels significantly elevated in the CSF, motor cortex and spinal cord of ALS patients, and multiple evidence indicated that the changes of CHIT1, CHI3L1 and CHI3L2 level in ALS patients were possibly associated with the disease severity and progression. These findings provided the novel clues for further understanding the ALS aetiology, developing immunomodulatory therapies and biomarkers for ALS [78].

ALS currently lacks the reliable diagnostic biomarkers. A meta-analysis evaluated CHIT1, CHI3L1, and CHI3L2 levels in CSF or blood and their diagnostic role in ALS patients. In ALS patients, the CHIT1 levels in CSF significantly elevated compared to controls with healthy control (HC), and CHIT1 levels elevated in the CSF of ALS patients compared to other neurodegenerative diseases (ONDS) control and exhibited an even more substantial increase when compared to ALS-mimicking diseases (AMDS). Similarly, the CHI3L1 levels in CSF were significantly higher in ALS patients compared to HC. CHI3L1 levels elevated in the CSF of ALS patients compared to ONDS and exhibited a more pronounced increase when compared to AMDS. The chitinases levels in the CSF of ALS patients showed a significant increase, supporting the chitinases role in CSF is as the diagnostic biomarkers for ALS [79].

## Diagnosis

Most neurologists still use the revised El Escorial criteria published in 2000 for the diagnostic classification of ALS [80]. The core elements of ALS diagnosis are the lesion that involves in four regions of the nervous system including the medulla oblongata, the cervical, thoracic and lumbosacral spinal cord. The diagnosis of ALS is divided into four levels according to the clinical symptoms, signs, and neurophysiological changes of UMNs and LMNs involvement in each regions of nervous systems. Some UMNs damaged signs must be located in the proximal of LMNs damaged signs. The laboratory-supported probable ALS must be the signs of UMNs and LMNs lesions at only 1 site, or UMNs signs at one site, plus the electromyography evidence of LMNs damage in at least two limbs. The probable ALS must be the signs of UMNs and LMNs lesions at only 1 site, or 2 or more UMNs signs, or LMNs signs located in the proximal of the UMNs damaged signs. The revised El Escorial diagnostic criteria in 2000 established a basic framework for the diagnosis of ALS with a good specificity but a poor sensitivity, especially for an early diagnosis, even some patients have not been diagnosed by the time of death yet. Therefore, this diagnostic criteria are more suitable for the clinical research and has a relatively poor clinical utility [81]. In response to the above diagnostic limitations, in 2015 the World Federation of Neurology revised the ALS diagnostic criteria again mainly for possible ALS in the diagnostic classification, which can be diagnosed without the evidence of UMNs damage when the clinical and neurophysiological evidence of LMNs damage is present in at least 2 sites and the relevant genetic testing has been done to exclude the specific types of ALS disease [82], this revised ALS diagnostic criteria are more practical for the clinical purposes, allowing the early diagnosis and early treatment of patients. However, it has been shown that the large proportion of ALS patients do not meet this revised El Escorial criteria until death yet, and that some patients with only LMNs symptoms can be found the pathological damaged signs in the corticospinal tract at post-mortem [83]. Before the 2015 revised El Escorial diagnostic criteria, in December 2006, researchers from the worldwide met in Awaji Island, Japan to discuss about the ALS diagnostic criteria and proposed the new ALS criteria called Awaji criteria in order to facilitate detecting ALS at the early stage of ALS. the Awaji criteria modified the early revised El Escorial criteria to further integrate the electrophysiological criteria with the clinical examination findings and added the presence of fascicular fibrillation potentials as the electrophysiological indicator of LMNs damage into the diagnostic criteria, the fascicular fibrillation potentials can replace the fibrillation potentials-positive sharp waves in the muscles of neurogenic damaged alterations [84]. Many studies after the release of the Awaji criteria have shown that the Awaji criteria increase the sensitivity of ALS diagnosis compared to the 2000 revised El Escorial criteria [85]. However, in the Awaji and the 2015 revised El Escorial diagnostic criteria, up to 50% of cognitive and behavioural changes in ALS patients are still not recognized. Therefore, the International Research Symposium on FTD and ALS held in London, Canada in June 2015, presented the revised consensus criteria for the diagnosis of frontotemporal lobe dysfunction in ALS patients [86], which increased the understanding of the neuropsychological characteristics about ALS patients. The understanding of the neuropsychological profile of patients with ALS have been considerable advances since the publication of the Strong diagnostic criteria in 2015.

**Figure 4.**
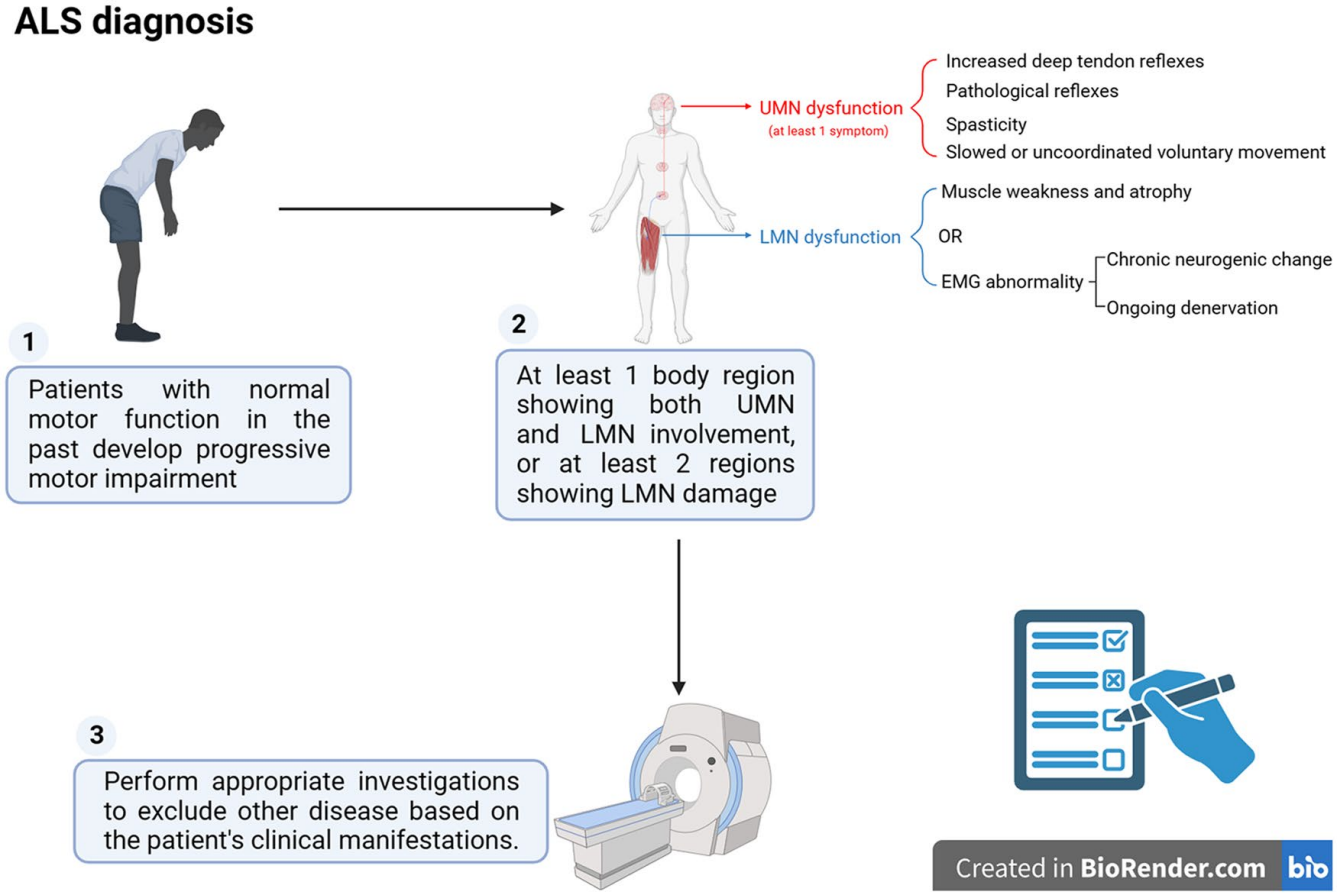
ALS diagnosis: according to the gold Coast criteria, the ALS diagnosis must fulfil 3 criteria. The UMNs dysfunction exhibits at least one symptom, including the increased tendon reflexes, the pathological reflexes, the increased velocity-dependent tone (spasticity) and the slowed or uncoordinated voluntary movement. The LMNs dysfunction exhibits the muscle weakness and atrophy or the electromyogram (EMG) abnormality which contains the chronic neurogenic change (increased duration and amplitude of motor unit potentials) and the ongoing denervation (fibrillation potentials or positive sharp waves, or fasciculation potentials). If body regions (bulbar, cervical, thoracic and lumbosacral spinal cord) exist the LMNs damage, it must be demonstrated that the different nerves and roots innervated 2 limb muscles, or 1 bulbar muscle, or 1 thoracic muscle appear abnormal *via* the clinical examination or EMG.

In order to further update the diagnostic criteria of ALS following the progression in the ALS studying, the International Federation of Clinical Neurophysiology, the World Federation of Neurology, the ALS Association and the Motor Neuron Disease Society were sponsored a meeting on the Gold Coast, Australia in September 2019 to propose a simpler set of ‘Gold Coast Criteria’ (Figure 4) [87]. Scholars involved in the development of the new criteria recognized that ALS involved more than the just motor system, the cognitive, behavioural and psychiatric disorders were also the part of the disease. The new diagnostic criteria not only presented the new methods for assessing the peripheral and central motor diseases, but also reviewed the current state of knowledge regarding the genetics of ALS. For the means assessing the peripheral and central motor disease processes, although the further research is needed, the recommendations for modifying the current criteria focus on how to simplify the diagnostic approach and how to create a single clinical diagnostic entity rather than identifying the different disease categories. The new criteria retain the concept of definitions of four nervous systems lesion regions, which is similar to the revised El Escorial criteria in 2015. In addition, the new criteria use a dichotomous approach, i.e. yes or no ALS, in the terms of classification, the new criteria are very similar to the Awaji criteria. The presence of both LMNs and UMNs dysfunction in just one nervous region is sufficient to meet the Gold Coast criteria. Studies have shown that the new criteria can be used in both clinical practice and scientific research [86].

Overall, the early diagnosis is important. For physicians, the encountering ALS patients at the onset of initial symptoms is critical for the timely recognition of the disease and the timely initiation of treatment. As the simplified diagnostic criteria become more commonly accepted, we anticipate that more physicians will recognize and treat ALS on the early course of the disease.

Overall, there is a 10-16 months delay in diagnosis from the onset of symptoms. There are a lot of factors involved in this delay. Studies have demonstrated that the ALS pathophysiological changes of motor neuron degeneration often occur before the onset of symptoms. At the pre-symptom stage, the genetic examination detected the certain common mutation genes such as *C9ORF72*, *SOD1*, *TARDBP*, *FUS* and *VCP* is sometimes helpful to identify fALS patients. However, approximately 90% of ALS patients are the sALS who have no genetic mutations. At the symptom onset stages, ALS can be diagnosed according to the signs of UMN and LMN degenerative damages through a clinical physical examination. The electrophysiological examination is very useful for the identification of LMN degeneration. At present, the most commonly used diagnostic criteria for ALS are the Awaji and the revised El Escorial criteria, which provides recommendations for ALS diagnosis partially based on the clinical signs of LMN and UMN degeneration. However, the clinical signs of UMN damage are covered because of the serious LMN damage destroys the reflex arcs. MRI can be used in detecting the morphological alterations in gray and white matter, but the MRI examination is very limited in that it does not allow for identifying the UMN damage such as the sclerosis signs of cortical spinal lateral tract and cortical brain stem tracts. Moreover, the clinical phenotypes of ALS vary extensively, which makes to be very difficult to differentiate ALS from the ALS-mimicking diseases, such as polyneuropathy, myopathy, sporadic inclusion body myositis, etc. at the disease super-early and early stages. However, it is estimated that total diagnostic timeline from the symptom onset to the confirmed diagnosis ranges from 8 to 15 months based on the contemporary diagnostic strategies including genetic testing and imaging, leading to a 10-16 months delay in diagnosis [88,89]. Because the median survival of ALS is only 20-48 months, this diagnostic timeline represents a significant proportion of the total disease duration and ultimately delaying the treatment and worsening the prognosis [89]. In general, the major factors leading to the ALS diagnostic delay mainly including the following several points: (1) Multiple specialists referral, the unnecessary testing and procedures/surgeries and misdiagnoses are a important factor leading to the diagnostic delay [90]. (2) Disease phenotype and diagnostic delay. The complexity of ALS clinical manifestations are an important factor in the timeline to decided diagnosis, particularly in the bulbar-onset versus spinal-onset ALS, it is reported that the delayed diagnostic timeline of bulbar-onset ALS patients is 3–7 months shorter than those of spinal-onset ALS patients due to the spinal-onset ALS patients have the mimic diseases of more differential diagnoses than those of bulbar-onset patients and are easier to be misdiagnosed. (3) Age of onset and diagnostic delay. The investigation results about this age-related diagnostic delay was not consistent. Some studies reported that the patients of over 60-year old were easier to be initially misdiagnosed compared to younger patients. However, in another study, it was reported that older patients showed a shorter diagnostic delay, showing a significant delay in the less than 45 years younger patients. Interesting, a study found that it was no association between age and the length of delay. (4) Gender and diagnostic delay. There is also the different investigation results in gender and diagnostic delay. Some investigations reported that the diagnostic delay of male ALS patients was longer than the female patients, which could be associated with the phenotype of bulbar-onset ALS is more in the female. In one study, it is found no significant difference between gender and diagnostic delay. (5) Patient comorbidities and diagnostic delay. ALS patients combining other neurological comorbidities, especially the ALS mimic clinical manifestations, is easier to occur the diagnostic delay [89].

## Treatment

### Drug treatment

**Figure 5.**
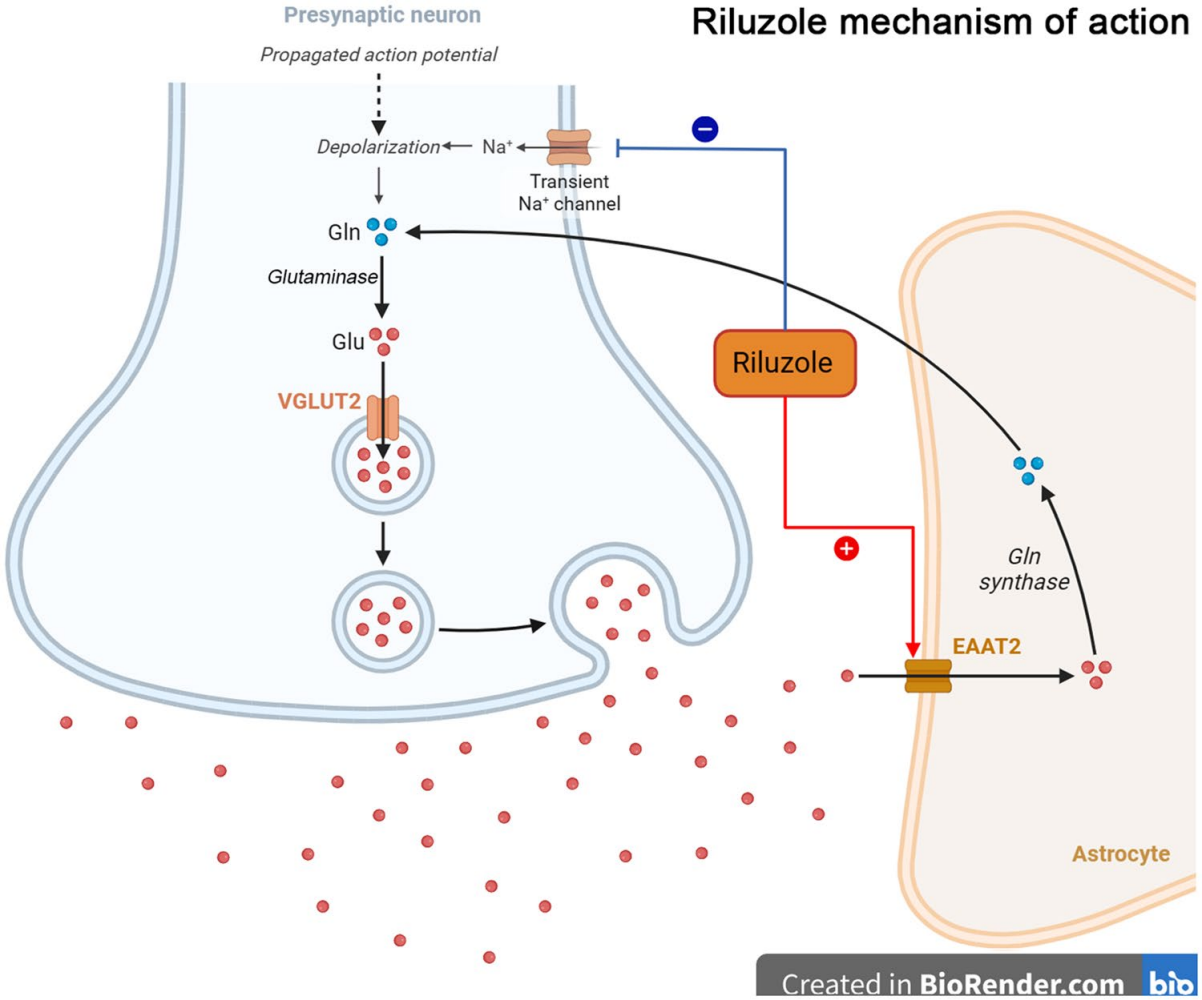
Riluzole mechanism of action: the astrocyte excitatory amino acid transporter 2 (*EAAT2*) expression is reduced in ALS patients, which may lead to excitotoxicity through reducing the glutamate clearance. On one hand, riluzole can reduce the presynaptic membrane glutamate release *via* the suppression of transient Na^+^ channels. On the other hand, riluzole can restore the EAAT2 expression in astrocytes and promote the glutamate reabsorption, thus reducing excitotoxicity. VGLUT2: vesicular glutamate transporter 2.

Riluzole is the first drug approved by both American Food and Drug Administration and European Union for the treatment of ALS. Its main mechanism of effect is to inhibit the toxic damage to neurons by glutamate neurotransmitter through various pathways [91]. For example, Riluzole can reduce the presynaptic membrane glutamate release by inhibiting the transient Na^+^ channels. On the other hand, Riluzole is also able to restore the expression of excitatory amino acid transporter 2 in astrocytes and promotes the glutamate reabsorption, thus reducing excitotoxicity, but Riluzole only partially delays the progression of ALS (Figure 5) [91,92]. A meta-analysis about the efficacy of the Riluzole treatment of ALS including 974 patients of Riluzole treatment and 503 ALS patients of no Riluzole treatment, showed that Riluzole resulted in a 9% increase in the probability of one year survival (49% in the placebo group and 58% in the Riluzole group) and an increase in the median survival from 11.8 months to 14.8 months compared to the control group, with small beneficial effects on both medulla oblongata and limb function [93]. In a long-term follow-up of sALS cohort, it showed that Riluzole was more effective in the elderly patients, those with the large body mass index and those with the higher ALS functional rating revised scores (ALSFRS-R) functional scores, and that the long-term Riluzole administration (> 6 months after diagnosis, cumulative dose >16,800 mg) was effective in improving prognosis [94].

A randomized, double-blind, placebo-controlled trial about the safety and efficacy of Edaravone in the treatment of ALS showed that patients with the definite or probable ALS met criteria for the disease duration of less than 2 years, the exertional spirometry (pulmonary function test) of more than 80%, and the ALSFRS-R subscale scores of more than 2, significantly smaller decreases in ALSFRS-R scores compared to controls [95]. Nevertheless, these trial designs may lack the generalizability to the broader population of patients with ALS, and another clinical trial did not find that the intravenous administration of Edaravone provided an additional clinically relevant benefit in a propensity score-matched cohort study [96]. No significant differences were found in both the disease progression and the respiratory function failure in patients with ALS treated with Edaravone in an Italian multicentre clinical trial [97]. Therefore, the use of Edaravone remains controversial and has not yet received a global approval [98].

The combination of dextromethorphan and quinidine was approved in US for the treatment of pseudobulbar affect, and the overall benefit of treatment was reflected in a reduction in the episodes of crying and laughing, as well as an improvement in the overall quality of life and the quality of relationships [99]. The drug is not available in all countries, but the alternative therapies and the more cost-effective treatments are available. The non-invasive ventilation also improves the survival and the quality of life in ALS patients [100]. Therefore, patients with ALS should be monitored regularly for the respiratory symptoms and receive the appropriate respiratory assessments such as the overnight oximetry or the partial pressure measurement of carbon dioxide, the blood bicarbonate concentration, spirometry, or the maximum inspiratory pressure to confirm their compliance with the non-invasive ventilation [101,102].

The new anti-inflammatory therapies targeting the immune system are also in clinical development. Phase 1 and 2 clinical trial results report that low doses of interleukin-2 are tolerated and the immune effective in increasing the number of regulatory T cells, although their impact on the progression of ALS is still being evaluated in phase 2b/3 trials [103]. The first autologous injection of expanded Treg cells was found to slow a disease progression in 2 patients [104]. Macitinib, a tyrosine kinase inhibitor reducing a microglia activation, has shown a effective promise in the clinical trials [105,106]. These reports highlighted the feasibility of immune-targeted drugs as the therapeutic candidates for ALS.

The effectiveness of currently approved drugs treated ALS is very limited, which urgently need to develop novel drugs. The preliminary studies demonstrated ultrahigh-dose methylcobalamin (injecting intramuscularly 50 mg dose methylcobalamin twice weekly for 16 weeks) to be a promising drug for treating ALS. The randomized clinical trial from 25 neurological centers in Japan suggested that ultrahigh-dose methylcobalamin was efficacious in slowing functional decline such as significantly improved the ALSFRS-R total score, the percent forced vital capacity, the noninvasive respiratory support, tracheostomy and ambulation. This therapeutic for the ALS patients of early-stage and moderate progression rate was safe and effect during 16 weeks treatment periods [107].

### Non-pharmacological treatment

In addition to drug therapy, researchers in various countries are actively seeking other treatments for ALS, including gene therapy and stem cell therapy. As the result of advances in ALS genetics research, the different approaches have been proposed to target the removal of known regulatory genes in ALS [108]. Four approaches are currently available to suppress the toxic effects of disease-causing genes. The use of microRNAs or antisense oligonucleotides (complementary DNA or RNA sequences) designed to pair with target sequences and activate RNA degradation. The use of small molecules to reduce the burden of mutant proteins (e.g. immune-mediated reduction), interference with transcriptional processes, and somatic mutagenesis, that is, reverse mutation to wild type in appropriate non-germ cells [109]. Gene therapy has shown some therapeutic effects in animal models, for example, SOD1 toxicity has been proven to damage MNs in the pathogenesis of ALS, so reducing mutant SOD1 protein expression may become one of the therapeutic methods for ALS, but it is still at the stage of animal experiments, which is still quite far from the clinical application [110]. Most of the gene variants that cause ALS are rare, therefore, there are few people who carry these variants, which will be a challenge in clinical trials like other innovative therapies.

Stem cell-based therapies for ALS and other neurodegenerative diseases have generated a great deal of interest over the past few years. Stem cell therapies are currently in phase 1, 2 or even 3 clinical trials using a variety of cell types. Current stem cell approaches are primarily designed to help protect the surviving MNs through paracrine effects (neuroprotection), they are not designed to replace the dead MNs. Mesenchymal stromal cells are widely used as the autologous stem cell therapy for ALS because of their ability to secrete neurotrophic factors and modulate the immune system, two mechanisms shown to slow the disease process in animal models [111]. In a phase 2 randomized controlled trial, the mesenchymal stem cell-neurotrophic factor cells were delivered intrathecally and intramuscularly to subjects with ALS, which showed the early promising signs of efficacy increased CSF neurotrophic factor and decreased CSF inflammatory biomarkers in the treated subjects, and the reduced progression rates of exertional spirometry and ALSFRS-R in the treated ALS patients [112,113]. Another stem cell strategy uses glial cell lineage precursors to treat ALS by growing stem cell lines from the foetal neural tissue and injecting them directly into the anterior horn of spinal cord [114]. Although not effectively powered, there appears to be a modest benefit in a subset of patients, and larger multicentre trials are being planned [115,116].

The treatments of ALS extend beyond pharmacotherapy and involve a comprehensive multidisciplinary approach. This approach has been shown to improve the quality of life and the survival of patients with ALS. It includes a team of the healthcare professionals such as neurologists, the physical and occupational therapists, nutritionists, the speech therapists, the respiratory specialists, the nurse practitioners, and the social workers or the palliative care teams. The multidisciplinary approach can improve disability and the quality of life by using ‘a problem-solving education process’ provided by the medical and allied health disciplines, for example, physiotherapy, the occupational therapy and the speech therapy, which focus on maximally attracting the activity and participation of ALS patients [117].

## Conclusions

In summary, ALS is a complex multifactorial disease, therefore, despite of some auxiliary diagnostic measures as well as the available of several medications and other treatments, the diagnostic value and treatment efficacy for ALS are very limited, therefore, ALS remains difficult to diagnose and treatment clinically. Due to the heterogeneous presentation and multiple phenotypes of ALS disease, as well as the overlap of signs and symptoms with other diseases, which bring a difficulty for early in the diagnostic process. The improved diagnostic criteria and the development of relevant ancillary tests could speed up the diagnosis and allow patients to earlier diagnose. Therefore, we anticipate that the future direction of clinical management of this disease will shift toward the simpler diagnostic criteria, such as the Gold Coast criteria. Many new therapeutic approaches for ALS are in development that promise to lead to more effective treatments in the near future. We anticipate that these research efforts will translate into the improved prognosis of the current and future patients with ALS. ALS is a progressive adult-onset neurodegenerative disease of the central nervous system, it is initially thought that this disease only damage MNs in both brain and spinal cord, the damaged regions were found more and more extensive accompanying with the deep investigation due to the progression of study technology. Currently, the investigated findings display that the complex ALS clinical manifestations involve cognition, behaviour and mental damage besides the signs and symptoms of motor system damage, which exhibits the multiply and variable clinical features and progression. Therefore, the clinical diagnosis of ALS become very difficult because of no special biomarker and special laboratory examination measures. Although the diagnostic criteria about ALS were revised several times, the current diagnostic criteria cannot accurately diagnosed ALS yet, especially at the early stage of ALS. Meanwhile, the efficacy of treatment drugs and measures of ALS are very limited yet although several drugs such as Riluzole, Edaravone and the dextromethorphan and quinidine combination as well as non-pharmacological treatment such as stem cells has been approved to use in the clinical treatment of ALS in lots of countries such as China, Canada and US. It is more pity that these treatments only slightly improve lifespan or symptoms, can’t cure and prevent of the progression of ALS. To this end, the clinical manifestations, diagnosis and treatment about ALS need more extensive and deep study and investigation in future.

## Data Availability

No data are available for this study.
